# *FOXL2* mutations in Chinese patients with blepharophimosis-ptosis-epicanthus inversus syndrome

**Published:** 2007-01-26

**Authors:** Juan Wang, Jinling Liu, Qingjiong Zhang

**Affiliations:** 1 State Key Laboratory of Ophthalmology, Zhongshan Ophthalmic Center, Sun Yat-Sen University, Guangzhou, China; 2 Eye Hospital, Zhongshan Ophthalmic Center, Sun Yat-Sen University, Guangzhou, China

## Abstract

**Purpose:**

Blepharophimosis-ptosis-epicanthus inversus syndrome (BPES) is an autosomal dominant disorder where eyelid malformation associated with (type I) or without (type II) premature ovarian failure (POF). It is ascribed to mutations in the forkhead transcriptional factor2 (*FOXL2*) gene. The purpose of this study is to identify mutations in *FOXL2* of Chinese patients with BPES.

**Methods:**

Genomic DNA was prepared from leucocytes of peripheral venous blood. The coding regions and nearby intron sequences of *FOXL2* were analyzed by cycle and cloning sequencing.

**Results:**

Four mutations in *FOXL2* were identified in six families, including c.241T>C, c.650C>G, c.804dupC, and c.672_701dup. Of the four, the c.241T>C and c.650C>G were novel and would result in missense changes of the encoded proteins, i.e., p.Tyr81His and p.Ser217Cys, respectively. The c.672_701dup (p.Ala224_Ala234dup) was detected in three families, indicating a mutation hotspot. The c.804dupC (p.Gly269ArgfsX265) mutation was found in one family.

**Conclusions:**

Our results expand the spectrum of *FOXL2* mutations and confirm the mutation hotspot in *FOXL2*.

## Introduction

Blepharophimosis-ptosis-epicanthus inversus syndrome (BPES, OMIM 110100) is a rare autosomal dominant disease with a prevalence of about 1 in 50,000 [1]. Clinically, BPES has been divided into two subsets depending on the association of ocular malformation with (type I) or without (type II) premature ovarian failure (POF) [2]. Genetically, however, both types are caused by mutations in *FOXL2*, and a genotype-phenotype correlation has been described in some cases [3,4].

The human *FOXL2* gene (OMIM 605597), located at 3q23, is a member of winged/forkhead transcription factor gene family [5]. This single-exon gene codes a protein with 376 residues, which consists of a DNA-binding forkhead domain (resudes 52-152) and a polyalanine domain (residues 221-234) [3,6,7]. A number of mutations in *FOXL2* have been identified [8], including six novel mutations in the Chinese population [9-11].

Here, we report four mutations identified in six Chinese families with BPES. Two novel missense mutations were associated with BPES type II.

## Methods

### Patients

Thirteen probands with BPES from unrelated families were collected from the Zhongshan Ophthalmic Center. Informed consent conforming to the tenets of the Declaration of Helsinki and following the Guidance of Sample Collection of Human Genetic Diseases (863-Plan) by the Ministry of Public Health of China was obtained from all participated individuals or their guardians prior to the study. The diagnosis of BPES was based on criteria previously established [12] with exclusion of microphthalmia.

### Mutation Analysis

Genomic DNA was prepared from leucocytes of peripheral venous blood [13]. Amplification of the genomic fragments encompassing *FOXL2* coding regions (NCBI human genome build 35.1, NC_000003 for gDNA, NM_023067 for mRNA, and NP_075555 for protein) was carried out by PCR using primers as follows: AF: 5'-CAG CGC CTG GAG CGG AGA G-3', AR: 5'-CTT GCC GGG CTG GAA GTG C-3', BF: 5'-GAC CCG GCC TGC GAA GAC A-3', BR: 5'-GGC CGC GTG CAG ATG GTG T-3', CF: 5'-CGC GGC CGC TGT GGT CAA G-3', CR: 5'-GCT GGC GGC GGC GTC GTC-3'. The sizes of the amplified DNA fragments are 545 bp, 517 bp, and 500 bp, respectively.

PCR amplification was carried out initially at 95 °C for 8 min, followed by 5 cycles at 94 °C for 40 s, at 68 °C for 40 s, at 72 °C for 40 s, then 5 cycles at 94 °C for 40 s, at 66 °C for 40 s, at 72 °C for 40 s, and a further 30 cycles at 94 °C for 40 s, at 64 °C for 40 s, at 72 °C for 40 s, and finally an elongation step at 72 °C for 5 min. Due to the high GC-rich nature of *FOXL2*, an additional 10% dimethylsulfoxide and 10% glycerol were added to the PCR mixture in order to successfully amplify the genomic fragments.

Direct sequencing of the PCR products was performed with an ABI BigDye Terminator Cycle Sequencing Kit v3.1 (ABI Applied Biosystem, Foster City, CA), using an ABI 3100 Genetic Analyzer. Sequencing results from patients as well as the *FOXL2* consensus sequences from the NCBI Human Genome Database (NC_000003) were imported into the SeqManII program of the Lasergene package (DNAStar Inc., Madison, WI) and then aligned to identify variations. Each mutation was confirmed by bidirectional sequencing. Mutation description followed the nomenclature recommended by the Human Genomic Variation Society (HGVS).

Any variation detected in *FOXL2* was further evaluated in available family members as well as in 100 normal controls by heteroduplex-SSCP analysis as described previously [14]. Two additional pairs of primers were used for heteroduplex-SSCP analysis. The sequence of these primers were: DF: 5'- CCG TAA GCG GAC TCG TGC-3', DR: 5'- AGT AGT TGC CCT TGC GCT C-3', EF: 5'- CGC ACT TCC AGC CCG GCA A-3', and ER: 5'- TGT GTA CGG CCC GTA CGA-3'.

In addition, one variation of insertions with multiple nucleotides that was found in three families was further analyzed by cloning sequencing. PCR products harboring this mutation were subcloned to pMD18-T Simple Vector (TaKaRa BIO, Japan) according to the manufacture's instructions. Clones with the mutant allele as well as the normal allele were selected by using heteroduplex-SSCP analysis. Sequence of the cloned fragment was identified by cycle sequencing as described above. Mutations were confirmed by sequencing three positive clones from each family. One mutation, c.241T>C, was further analyzed by PCR-RFLP analysis since the mutation creates a new *FOK*I site.

## Results

**Figure 1:**
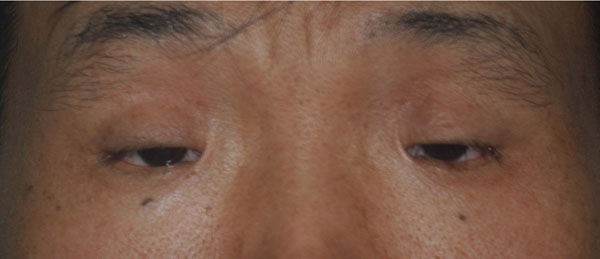
Clinical phenotype of a Chinese patient with BPES. A photo of the eyelid area from a 40 years old man (III:1 from family E in Figure 3) demonstrated typical phenotype of BPES. The horizontal diameter of his cornea was 11.5 mm for both eyes. Ultrasound A-scan recorded an axial length of 24.19 mm/OD and 24.43 mm/OS. His refractive measurement was -3.00DCX180°/OD and -4.50DCX180°/OS.

**Figure 2:**
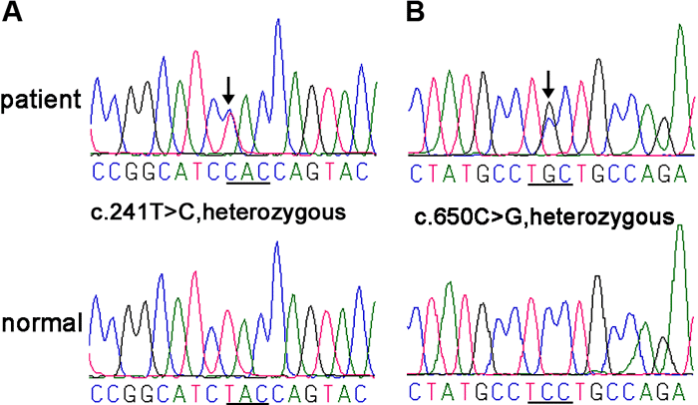
Sequencing results of the two novel mutations in *FOXL2.* Sequence chromatograms of Family A (**A**) and family B (**B**), and their corresponding normal sequences. The underline below each sequence highlights the codon containing the mutation.

**Figure 3:**
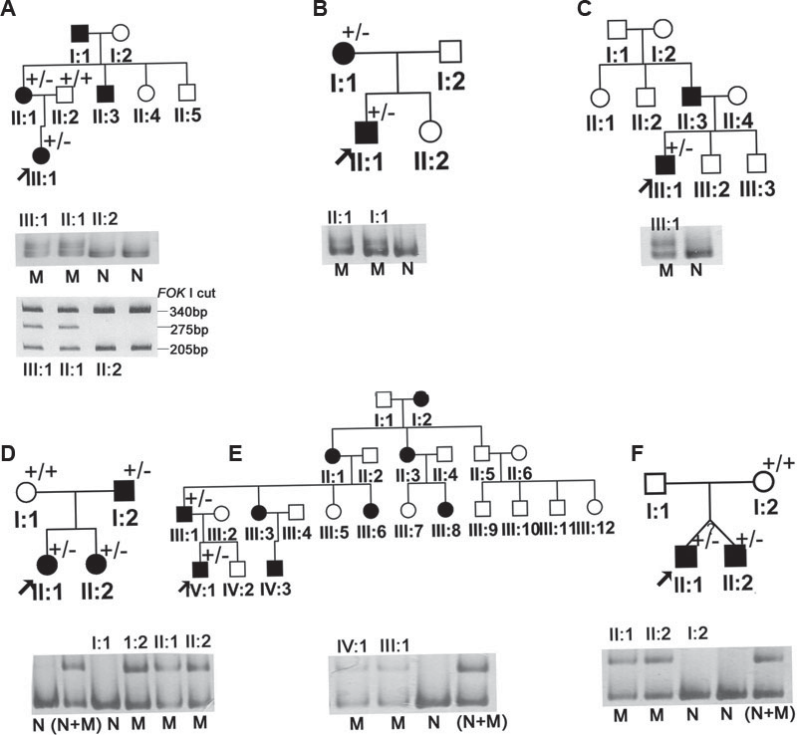
Pedigrees and heteroduplex-SSCP analysis. Pedigrees of the different families (A, B, C, D, E, and F) are shown. Black filled symbols indicated patients affected with BPES in each family. The "+/+" or "+/-" sign indicated individuals analyzed with normal sequences or heterozygous mutation in *FOXL2*, respectively. The "M" under each lane indicated mutation, the "N" represented normal individuals, and "N+M" represented a mixture of PCR products resulted from normal and mutant clones. In addition, results of FOKI digestion for family A were present at the bottom of Figure 1A. The c.241T>C mutation in family A creates an additional *FOK*I site. By *FOK*I digestion of the 545 bp PCR product, normal allele yielded two fragments (340 bp and 205 bp), but the mutant allele yielded three fragments (275 bp, 205 bp, and 65 bp). Patients with the heterozygous c.241T>C mutation had four bands (only three bands were shown in the figure) as compared to normal individuals with two bands.

All patients demonstrated typical features of BPES, including small palpebral fissures, ptosis of the eyelids, and epicanthus inversus (Figure 1). Upon complete sequencing analysis of *FOXL2* for 13 probands with BPES, four heterozygous mutations were found in six probands, including c.241T>C, c.650C>G, c.804dupC, and c.672_701dup (Figure 2; Table 1). Of the four, c.241T>C and c.650C>G are novel. All four heterozygous mutations were further detected by heteroduplex-SSCP analysis, and one (c.241T>C) was further detected by *FOK*I digestion (Figure 3). These mutations were also present in affected patients from corresponding families but neither in unaffected individuals nor in 100 controls.

**Table 1:** *FOXL2* mutations detected in Chinese patients with BPES.

**Family**	**DNA change**	**Mutation type**	**Location**	**Protein change**	**BPES**
A	c.241T>C	Missense	Forkhead	Tyr81His	Type II
B	c.650C>G	Missense	Immediately upstream polyalanine	Ser21Cys	Type II
C	c.804dupC	Insertion	Downstream of polyalanine	Gly269ArgfsX265	Unknown
D	c.672_701dup	Duplication	Polyalanine domain	Ala224_Ala234dup	Unknown
E	c.672_701dup	Duplication	Polyalanine domain	Ala224_Ala234dup	Type II
F	c.672_701dup	Duplication	Polyalanine domain	Ala224_Ala234dup	Unknown

Subtypes of BPES in families C, D, and F are unknown, as there were no female patients (families C and F) or the female patients were too young (family D, where the two female patients were only 4 or 2 years old, respectively).

**Figure 4:**
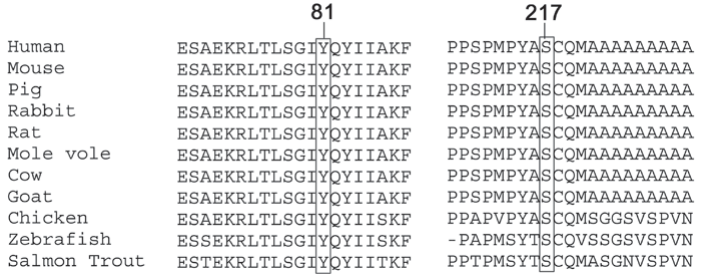
Multiple alignment of 11 FOXL2 orthologs. This demonstrated high conservation of residues involved by the p.Tyr81His and p.Ser217Cys mutation. The resource for the 11 FOXL2 orthologs was as follows: human (*Homo sapiens*, NP_075555), mouse (*Mus musculus*, NP_036150), pig (*Sus scrofa*, AAQ91845), rabbit (*Oryctolagus cuniculus*, AAQ91846), rat (*Rattus norvegicus*, XP_345976), mole vole (*Ellobius lutescens*, AAV30684), cow (*Bos taurus*, NP_001026920), goat (*Capra hircus*, AAM52099), chicken (*Gallus gallus*, NP_001012630), zebrafish (*Danio rerio*, XP_698915), and salmon trout (*Oncorhynchus mykiss*, AAS87040).

The c.241T>C (p.Tyr81His) mutation results in substitution of a charge-free tyrosine with a charge-positive basic hydrophilic histidine within the forkhead domain. The c.650C>G (p.Ser217Cys) mutation is located immediately upstream of the polyalanine domain. The tyrosine at position 81 and the serine at position 217 are well conserved in *FOXL2* by ClustalW analysis of 11 orthologs from related vertebrate species (Figure 4).

## Discussion

*FOXL2* encodes a forkhead transcription factor containing a forkhead domain for DNA-binding and a polyalanine domain of uncertain function. Strong expression of *FOXL2* has been found in eyelids [3,15], developing periocular muscles, and surrounding tissues [16,17]. Of the four mutations identified in this study, the c.241T>C affected the forkhead domain, while the other three (c.650C>G, c.804dupC, and c.672_701dup) were located upstream, within, and downstream of the polyalanine domain, respectively.

Missense mutations in *FOXL2* reported so far usually occurred at the forkhead domain [9,17-19], except two, such as c.650C>T in a Belgian family [4] and c.644A>G in a five-generation family from south-India [20]. The clinical subtypes of the patients with the c.650C>T and c.644A>G mutations were unknown. The novel c.650C>G (p. Ser217Cys) mutation identified in Chinese family B occurred at the same site as that found in the Belgian family, which is located immediately upstream of the polyalanine domain. The serine at position 217 is well conserved in 11 orthologs (Figure 4). It has been shown that mutations affecting the polyalanine domain induce extensive nuclear and cytoplasmic protein aggregation [21,22]. Missense changes have been suggested to act as null allele leading to BPES phenotype due to haploinsufficiency [4] or dominant-negative effect [20,23].

It has been suggested that *FOXL2* mutations truncating the protein led to BPES type I while those extending the mutant protein were associated with type II [3,4]. However, intra- and inter-family phenotypic variations have been found [3,4,19,24,25] so that this genotype-phenotype correlation might not be general [18,19,26]. The c.804dupC mutation has been shown to cause both types of BPES [4,19,25], and the c.672_701dup causing polyalanine expansion most likely leads to BPES type II [19]. Missense mutations have been associated with both BPES type I [17] and II [3,19]. The patients from families A and B in this study, with novel c.241T>C and c.650C>G mutations, respectively, had type II BPES. The c.650C>G mutation is the first mutation described that occurs immediately upstream of the polyalanine domain and associated with type II BPES. This may raise a possibility that the region containing the c.650C>G mutation is of importance for *FOXL2* function.

The c.672_701dup (p.Ala224_Ala234dup) was found in families D, E, and F (Table 1), consistent with a mutation hotspot. To check the origin of the c.672_701dup mutation in three families (families D, E, and F in Figure 3), six SNPs (including rs13325788, rs2291252, rs28937885, rs7432551, rs28937884, and rs11924939) were analyzed (Table 2). The SNP at rs2291252 is different between patient II:1 from family D and patient III:1 from family E, which may suggest a different origin of the mutant allele. The mutation in family F is most likely a de novo event as BPES was not present in the patients' parents although the SNPs in the patient II:1 in family F were the same as that of II:1 in family D. It has been reported that 30% of the *FOXL2* mutations lead to polyalanine expansion [19]. The c.672_701dup has been found in BPES families of Caucasian [4,19,27,28] and Asian origin [10,29].

**Table 2:** Results of SNPs analysis of the three families with the c.672_701dup mutation.

**Family**	**Patient**		**Upstream of FOXL2**	**Inside FOXL2**			**Downstream of FOXL2**	
			**rs11924939**	**rs28937885**	**rs7432551**	**rs28937884**	**rs13325788**	**rs2291252**
d	II:1	C		T	C	T	G	C
e	III:1	C		T	C	T	G	T
f	II:1	C		T	C	T	G	C

The origin of the c.672_701dup mutation in family D is different from family E as they have different SNP at rs2291252. This mutation in family F is a de novo event, although patient II:1 in family F shares the same haplogroup of the six SNPs with that of family D.

In summary, we identified two novel and two known mutations in *FOXL2* of six Chinese families with BPES. The two novel mutations are the first reported instances that were associated with BPES type II. Our results expanded the spectrum of *FOXL2* mutations and confirmed the mutation hotspot in *FOXL2*.
